# Complementary Effects of Two Growth Factors in Multifunctionalized Silk Nanofibers for Nerve Reconstruction

**DOI:** 10.1371/journal.pone.0109770

**Published:** 2014-10-14

**Authors:** Tony M. Dinis, Guillaume Vidal, Rodrigo R. Jose, Pascale Vigneron, Damien Bresson, Vincent Fitzpatrick, Frédéric Marin, David L. Kaplan, Christophe Egles

**Affiliations:** 1 Université de Technologie de Compiègne, CNRS UMR 7338: BioMécanique et BioIngénierie, Centre de Recherches, BP 20529, Compiègne, France; 2 Department of Biomedical Engineering, Tufts University, Medford, Massachusetts, United States of America; 3 Department of Oral and Maxillofacial Pathology, Tufts University, School of Dental Medicine, Boston, Massachusetts, United States of America; Université de Lorraine, France

## Abstract

With the aim of forming bioactive guides for peripheral nerve regeneration, silk fibroin was electrospun to obtain aligned nanofibers. These fibers were functionalized by incorporating Nerve Growth Factor (NGF) and Ciliary NeuroTrophic Factor (CNTF) during electrospinning. PC12 cells grown on the fibers confirmed the bioavailability and bioactivity of the NGF, which was not significantly released from the fibers. Primary neurons from rat dorsal root ganglia (DRGs) were grown on the nanofibers and anchored to the fibers and grew in a directional fashion based on the fiber orientation, and as confirmed by growth cone morphology. These biofunctionalized nanofibers led to a 3-fold increase in neurite length at their contact, which was likely due to the NGF. Glial cell growth, alignment and migration were stimulated by the CNTF in the functionalized nanofibers. Organotypic culture of rat fetal DRGs confirmed the complementary effect of both growth factors in multifunctionalized nanofibers, which allowed glial cell migration, alignment and parallel axonal growth in structures resembling the ‘bands of Bungner’ found *in situ*. Graftable multi-channel conduits based on biofunctionalized aligned silk nanofibers were developed as an organized 3D scaffold. Our bioactive silk tubes thus represent new options for a biological and biocompatible nerve guidance conduit.

## Introduction

When a peripheral nerve is sectioned, the axonal segment distal to the lesion site undergoes Wallerian degeneration [1], [2], while the proximal segment is able to regenerate axonal sprouts that can eventually re-establish motor and sensory nerve function. However, the repair of peripheral nerve lesions, especially after the loss of a full segment of the nerve, remains a challenge in reconstructive surgery [3]. Clinical strategies to treat peripheral nerve injuries include the use of a primary suture in an end-to-end manner, the use of a biological nerve guide such as arteries, veins or muscles for small gaps and the use of nerve autografts for larger gaps [4]. However, autologous nerve transplantation remains inadequate for large and complex lesions of peripheral nerves because of the limited availability of materials suitable for grafting and donor site morbidity [5].

Recent years have witnessed the development of increasingly sophisticated bioengineered nerve graft substitutes composed of synthetic or natural polymer-based nerve guides. These biodegradable nerve guides have been prepared with or without nerve growth factors or with pre-seeded Schwann cells [6], [7]. The ability of the scaffold to support axonal growth and also to stimulate and direct this response is pivotal in promoting efficient nerve tissue repair [8], [9].

Silk is a well-known material used for centuries in the textile industry and for decades by physicians as sutures. Silk is a natural macromolecular protein polymer which is synthesized by epithelial cells of specialized glands, is then secreted into the lumen and finally is spun into fibers by several Lepidopteran larvae [10]. The sericin-free silk fibroin fiber exhibited excellent biocompatibility both in vitro and in vivo [11]. Over the last few years, silk fibroins (SF) have been increasingly studied for biomedical applications due to their favorable biocompatibility and controllable degradation rates, which can be tuned from hours to years based on the mode of processing. SFs have also demonstrated remarkable mechanical properties and the ability to be shaped into different material formats [12].

One of the major challenges remaining in regenerative neurobiology is to design suitable biomimetic nerve guides. In order to achieve the desired functionality of the nerve that is to be replaced, the guide needs to be carefully engineered to elicit specific responses from local cells and tissues [13]. Many techniques have been developed for fabricating fibrous scaffolds for use as tissue substitutes. Electrospinning has emerged a useful technique due to the ability to generate fibers similar to the scale of fibrous structures of native extracellular matrices (ECM) [14]. In addition, electrospinning is a relatively simple, robust and versatile technique capable of generating fibers with diameters in the nanometric range [15]. Bioactive molecules such as adhesive molecules, biomimetic peptides and growth factors have been incorporated into the fibers upon spinning to stimulate the proliferation and differentiation of seeded cells during in vitro culture [16], [17]. Among the many growth factors found in the body, neurotrophic factors (NTFs) play a crucial role in regulating the development and function of different sets of neurons of the mammalian nervous system [18]. Nerve Growth Factor (NGF), for example, promotes neuronal survival and regeneration both in vitro and in vivo [19]. Another neurotrophic factor, the ciliary neurotrophic factor (CNTF) produced by astrocytes and abundantly present in peripheral nerves, is localized mainly in the cytoplasm of myelinating Schwann cells and its synthesis is decreased during Wallerian degeneration [20], [21]. It has also been shown that CNTF is a neuroprotective agent and has improved the remyelination of regenerated nerves [22], [23].

In the present study we aimed at developing new multifunctionalized silk-based material to support and promote peripheral nervous system (PNS) nerve regeneration. To emulate the preclinical development of this material, the material system was evaluated using: (i) a neurotrophic sensitive cell line, (ii) rat dorsal root ganglia primary neuronal cultures and (iii) an organotypic culture of the entire rat dorsal root ganglia. This multiscale strategy supported the demonstration of the full capacities of this new silk-based nanomaterial system, leading to the synthesis of a biological, biodegradable and bioactive 3D nerve guide for surgical implantation.

## Materials and Methods

### 1 Materials

Cocoons of the silkworm *Bombyx mori* were supplied by Tajima Shoji Co. (Yojohama, Japan). All Chemicals were supplied by Sigma-Aldrich, Inc. (St. Louis, MO, USA). Slide-a-Lyzer dialysis cassettes were purchased from Pierce, Inc. (Rockford, IL, USA). All products for cell culture were from Gibco, Life Technologies SAS, France. Growth factors, NGF and CNTF were supplied by R&D Systems, Inc. (Minneapolis, MN, USA).

### 2 Preparation of Silk fibroin solution

Silk solutions were prepared following our previous published procedures [24]. In brief, *B. mori* silkworm cocoons were cut into small pieces and boiled for 30 minutes in an aqueous solution of 0.02 M Na_2_CO_3_, rinsed for 20 min three times with cold deionized (DI) water, and then allowed to dry for 48 h at room temperature. Dried silk fibroin was dissolved in 9.3 M LiBr solution at 60°C for 4 h. Silk solutions were dialyzed against DI water using Slide-a-Lyzer dialysis cassettes (membrane MWCO 3500) for 72 h to remove salts. The final concentration of the aqueous silk fibroin solution was 6–7 wt%, which was calculated by weighing the remaining solid after drying. The solution was then concentrated by dialyzing against 15% (w/v) polyethylene glycol (PEG) for 5 h to produce ∼10 wt%. Silk solutions were stored at 4°C.

### 3 Preparation of spinning functionalized solution

Silk fibroin solutions were mixed with 5 wt% polyethylene oxide (PEO, Mw =  900,000) in a 4∶1 ratio to produce a 9% silk/PEO solution. To functionalize the spinning solution, NGF, CNTF or both growth factors, reconstituted in PBS containing 0.1% bovine serum albumin solution, were added to the spinning solution at a concentration of 1 µg.mL^−1^ and 0.1 µg.mL^−1^, respectively.

### 4 Electrospinning

Electrospinning was performed according to procedures described previously [17]. In brief, silk fibroin solution (functionalized or not) was delivered through a 16G stainless-steel capillary at a constant flow rate of 0.015 mL.min^−1^ with a syringe pump (Thermo Scientific, Waltham, MA, USA). The syringe needle was set at a voltage of 12 kV with a high voltage power supply (Gamma High Voltage Research ES-30P, Ormond Beach, FL, USA) and mounted at the center of a 10 cm diameter aluminum plate. The distance between the needle and the ground collector was set to 15 cm. Aligned silk fibers on glass cover slips were produced using an oscillating ground deposition system (ODS) consisting of switching an electrical ground back and forth between two flat, zinc-plated steel plates which were used as collectors. The mechanical switch (McMaster-Carr, Inc., Atlanta, GA, USA) alternates the location of ground between two independent collectors and was maintained continuously during the electrospinning process at 0.33 Hz [25]. Glass cover slips were deposited on tape to collect the electrospun silk fibroin. For 3D scaffold design, the electrospinning duration was 90 min and the collector consisted of a rotating mandrel (rotation speed: 10 m.s^−1^). All electrospun fibroin were treated by water vapor annealing for 8 h to obtain water insoluble silk mats [26].

### 5 Nanofibers diameter and alignment

More than 200 Fibers were measured using an image processing software (ImageJ, MATLAB). Fiber diameters were measured from SEM images of the aligned scaffold. Straight lines were drawn along the length of the image and at each intersection of lines and electrospun fibers. The diameter and the angle of each fiber were calculated using ImageJ to define nanofiber alignment.

### 6 Roughness measurement

The Zygo NewView 200 is a scanning white-light interferometer that uses Frequency Domain Analysis (FDA) to generate quantitative 3D images of surfaces [27]; measurements use a white light filter based on a center wavelength of 600 nm, with a bandwidth of 125 nm. The interference patterns were recorded by a CCD camera; each measurement contains 320 × 240 data points.

### 7 ELISA

In order to prevent any release of loosely bound factors NGF and CNTF, functionalized electrospun nanofibers (n = 3) were incubated in 2 mL of sterile PBS containing 0.1% BSA at 37°C without any pre-treatment (like ethanol sterilization) and 150 µL were harvested after 1, 2, 8, 48 and 120 hours and stored at −80°C. NGF and CNTF release assays were performed with the ChemiKine Nerve Growth Factor Sandwich ELISA Kit (Millipore, France) and Raybio Rat CNTF ELISA kit (TEBU-BIO, France) respectively, according to the manufacturer's instructions.

### 8 PC12 cell culture

The PC12 cell line was a generous gift from Dr Frédérique René (Faculté de medicine, Strasbourg, France). All products are from Gibco, except rat NGF from R&D systems, France. Cells were routinely cultured in RPMI 1640 medium complemented with 10% horse serum, 5% decomplemented fetal bovine serum, 2 mM L-glutamine, 100 U.mL-1 penicillin and 100 µg.mL-1 streptomycin and subcultured once a week at 25,000 cells/cm^2^ after trypsinisation, with medium changes every 2–3 days. For differentiation assays, cells were seeded at 10,000 cells/cm^2^ in differentiation medium composed of RPMI 1640 complemented with 1% horse serum, 2mM L-glutamine, 100 U.mL^−1^ penicillin and 100 µg.mL^−1^ streptomycin. The medium was renewed every 2–3 days. Cells were incubated for 7 to 11 days at 37°C in an atmosphere of 95% air and 5% CO2. For positive controls, 50 ng.mL^−1^ of rat NGF was added in differentiation medium. Statistical analysis was performed using InStat software (Software Inc., GraphPad, San Diego, CA). Values were expressed as mean ± standard deviation (mean ± SD). Neurites per cell and neurites lenght from each group were compared using nonparametric ANOVA (Kruskal-Wallis Test and Tukey post hos test). A p-value less than 0.05 was considered to be significant.

### 9 DRGs extraction and cells culture

DRG cells were obtained from young male Sprague Dawley rats (1–3 months old) euthanized with pentobarbital sodium. All procedures were carried out in compliance with the Ethical Committee and the Veterinary Authorities of France in accordance with the European Communities Council Directive of 22 September 2010 (2010/63/UE). This study was specifically approved by the Université de Technology de Compiègne ethics committee. DRGs were extracted under hood and incubated in 2.5% collagenase (Sigma) for 15 min at 37°C and pellet by centrifugation (1 min 500g). They were incubated 15 min in trypsin (Sigma) 0.5% and DNase I (Sigma) at 1 mg.mL-1 at 37°C and pellet by centrifugation (1 min 500g). After a wash with F-12 medium, DRGs were dissociated by ten passages in a fire-polished Pasteur pipette. The mean yield of cells obtained per rat was 100,000 with 90% viability. Cells were seeded at 7,500 cells per glass slide (12 mm square). For organotypic culture, DRGs were extracted from E15 rat embryos. Finally, glass slides were coated with poly-lysine/laminin before seeding, as described in Malin et al. [28]. For cells and organotypic culture, medium consisted in 37°C pre-warm F-12 medium (Gibco), 10% horse serum (Gibco), 100 U.mL-1 penicillin and 100 µg.mL-1 streptomycin. Medium was changed every 3 days.

### 10 Scanning electron microscopy

Samples were rinsed in PBS, fixed in 3% glutaraldehyde in Rembaum buffer (pH 7.4) [29] for 1 h, dehydrated in a series of graded alcohols, critical-point dried from CO_2_ (Polaron Instrument Inc., Nottingham UK), sputter-coated with gold (Polaron) and examined in a Philips ESEM-FEG XL30 environmental scanning electron microscope (ESEM).

### 11 Immunofluorescence imaging

Cells and DRGs were fixed at different times post-seeding in 4% paraformaldehyde solution in PBS for 15 min and 45 min respectively at room temperature and rinsed three times with PBS. Samples were permeabilized in 0.5% Triton X-100 in PBS for 5 min at room temperature and rinsed twice with PBS before 1 h incubation at room temperature in 1% bovine serum albumin (BSA) in PBS. Rabbit primary antibody directed against βIII tubulin (Sigma-Aldrich, France) was diluted (1∶150) in PBS/BSA 0.1% (w/v) and incubated at room temperature for 1 h. The cells were washed two times with 0.1% BSA in PBS. Cy3-conjugated secondary antibody (cy3 goat anti-rabbit IgG, Jackson Immunoresearch), 40,6-diamidino-2- phenylindole (DAPI; 1 µg.mL^−1^, Sigma) and Phalloidin-X5- 505 (0.16 nmol ml^−1^, Fluoroprobes, France) were added and incubated at room temperature in the dark for 1 h. The samples were then rinsed with PBS, mounted in Mowiol and observed using a LEICA DMI 6000 microscope or a LSM 710 scanning confocal microscope which images were analyzed with ‘Zen 2011’ software.

### 12 Morphometric and statistical analysis

Images were analyzed using Mathworks' Matlab software. Low signal was filtered out using a threshold based on intensity (the silk fibers and background noise present lower pixel intensities than the cell bodies and neurites). In each image, the number of cells was determined using the signal from DAPI staining. Then each neurite was isolated using the tubulin staining and its length was measured using the ‘bwmorph’ and ‘regionprops’ functions of the Matlab Image Processing Toolbox. This step was carried out by skeletinization of the neurite, and if necessary manual cropping of superfluous branches. Artifacts and signal from cellular debris were eliminated from analysis using the ‘bwareaopen’ function of the Matlab Image Processing Toolbox. The results were interpreted by determining the average number of neurites per cell and the average neurite length.

For the average number of neurites per cell, average neurite length and glia/neurons ratio data ± SEM from 3 independent experiments were analyzed for each condition and at least 90 cells have been analyzed per condition. Statistical analyses were performed with ‘InStat’ software, using the Mann-Whitney unpaired with two-tailed P test.

### 13 3D silk tube

After 90 min. of electrospinning, electrospun fibers were removed from the mandrel and rolled up at 360° about 40 times using Teflon sticks (0.3 mm diameter) in order to get a final tube of about 1.5 mm diameter (close to that of a rat sciatic nerve). Teflon sticks were removed after the water vapor annealing process. The obtained silk tube was wetted overnight at 4°C in sterile PBS and then sutured (10-0 suture FG 2850; Ethicon, Somerville, NJ) to a sciatic nerve of a Sprague-Dawley male rat (2 months old).

## Results

### 1 Electrospinning setup and nanofiber alignment

**Figure 1:**
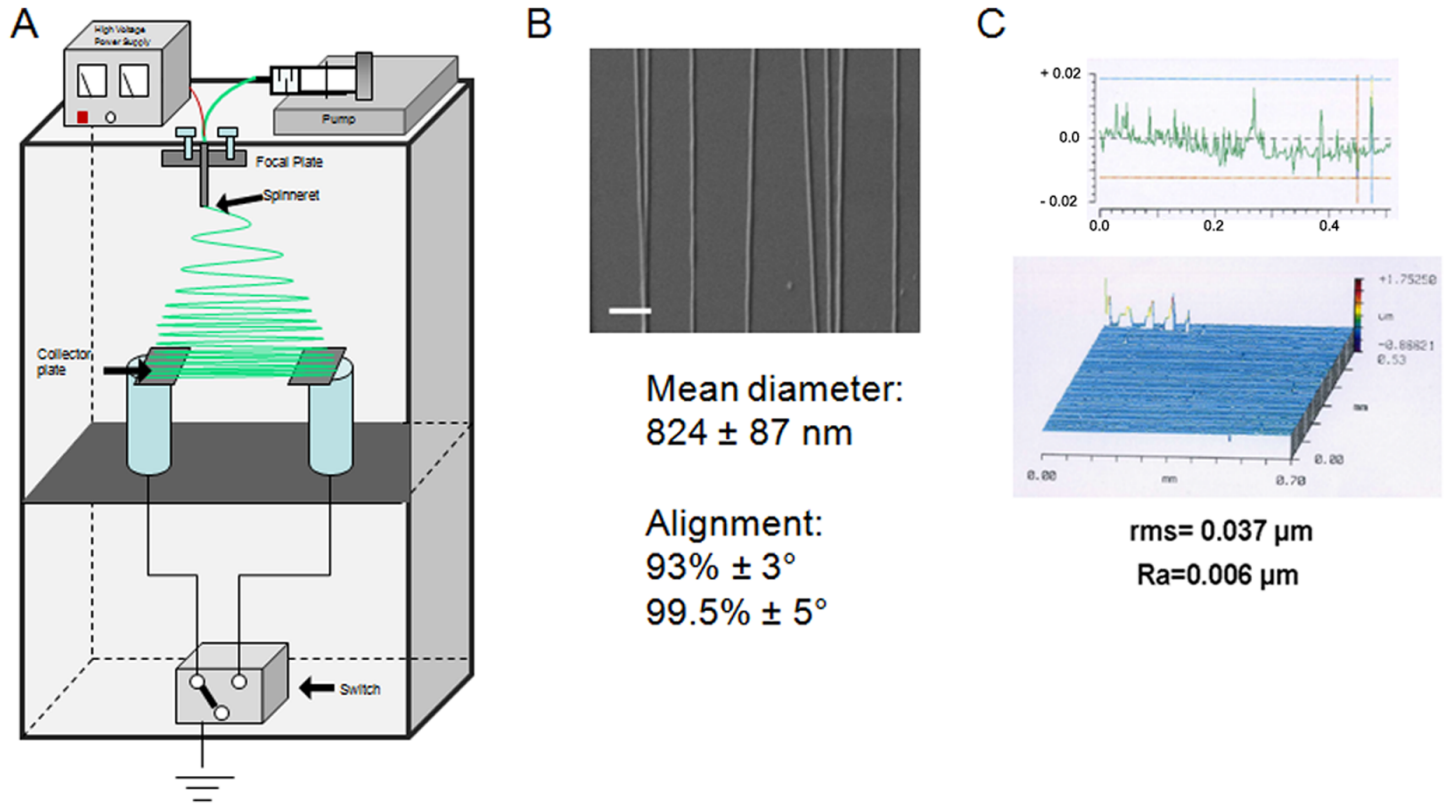
Fabrication and characterization of biofunctionalized and aligned silk nanofibers. A) Fibroin solution was mixed (or not) with NGF and CNTF and electrospun as described in material and methods. B) SEM image of aligned silk nanofibers on glass slides; Alignment and mean diameter of electrospun fibers, measured as described in material and methods; Scale bar: 5 µm. C) Oblique plot and surface profile of fibroin nanofibers electrospun on a glass slide.

The collector consisted of two pieces of conductive substrates which were separated by electrical tape with glass slides collecting nanofibers (Figure 1A). One of the key features with this technique is that it is convenient to transfer well-aligned fibers onto other solid substrates for further utility, such as glass slides for cell and organotypic culture. The electrospinning settings lead to uniaxially aligned nanofibers with an average diameter of 854 ± 87 nm (Figure 1B and C). Also, 99.5% of the fibers present an angle of deviation less than 5°. The duration of the electrospinning process was adjusted to obtain a low roughness of nanofibers on the glass slide with a root mean square (RMS) value of 0.036 µm and Ra of 0.007µm (Figure 1C).

### 2 Nanofiber functionalization and bioactivity

**Figure 2:**
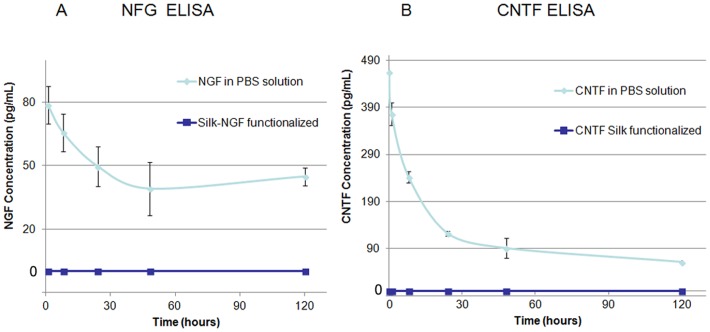
Growth factors release from electrospun functionalized silk and stability. A) No NGF was detected from the silk electrospun after 5 days in PBS. B) As NGF, no CNTF was detected from the electrospun. Degradation of growth factors was observed in PBS with 0.1% BSA. After 5 days, more than 50% of NGF could be detected unlike 10% for CNTF.

NGF (1 µg.mL^−1^) and CNTF (0.1 µg.mL^−1^) were added to the spinning solution to functionalize nanofibres. The release of NGF and CNTF from fibroin nanofibers after electrospinning was monitored by ELISA at different time points after sample incubation in PBS. For each batch of neurotrophic factors, one group contained soluble NGF (80 pg. mL^−1^) or CNTF (400 pg. mL^−1^) as a positive control. The detected amount from these controls decreased rapidly for NGF (37% after 24h and 50.1% after 48h) and even faster for CNTF (74% after 24h and 80.5% after 48h), suggesting rapid degradation of these neurotrophic factors. NGF and CNTF release was not detected from the nanofibers over 4 days, suggesting that both growth factors were trapped, strongly adsorbed to the nanofibers or released below the detection level of the ELISA kit (Figure 2 A and B).

**Figure 3:**
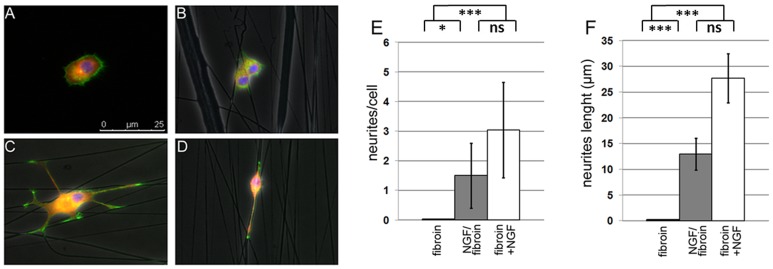
PC12 differentiation induced by NGF-functionalized nanofibers. PC12 cells grown for 8 days on: glass slide a), fibroin nanofibers b), fibroin nanofibers with NGF (50 ng. mL-1) in culture medium c) and NGF-functionalized fibroin nanofibers d); scale bar: 25µm. Number of neurites per cell e) and neurites length f). White bars: NGF in solution, grey bars: NGF-functionalized fibroin and black bars: fibroin. Quantification was performed as described in material and methods. Standard deviations are shown as error bars. Significant differences (**ns**: no significance, * p<0,05; *** p<0,001)

To assess the bioactivity of the immobilized NGF, PC12 cells were grown on the functionalized nanofibers. PC12 cells were provided as a gift by Dr. Frédérique René (INSERM 1121, France), cells originated from the ATCC cell bank (Manassas, VA, USA). PC12 is a cell line derived from a pheochromocytoma of the rat adrenal medulla. PC12 cells stop dividing and terminally differenciate when they are treated with NGF [30]. This response makes PC12 cells a useful system for neuronal differentiation as well as a good ‘biological sensor’ of NGF availability in fibroin nanofibers [31]. When cultivated on glass slides without NGF, the cells were round and showed an undifferentiated morphology with no cytoplasmic extensions (Figure 3A). The culture of PC12 cells on electrospun silk nanofibers (no growth factors) did not result in any change in morphology (Figure 3B), whereas neurite-like extensions were evident on the cells grown on silk nanofibers with NGF in culture medium (50 ng.mL^−1^, Figure 3C). These cells had an average of 3 neurites per cell with an average length of 27.7 µm, and the longest neurite about 40 µm long (Figure 3E and F), confirming the differentiation of PC12 cells induced by NGF. These neurite-like structures extended without any particular direction and seemed to adhere to the nanofibers. When cultured on NGF-functionalized fibroin nanofibers without any NGF in the culture medium, the cells presented neurite-like structures confined to the NGF-functionalized fibers (Figure 3D). These cells had an average of 1.5 neurites per cell with a mean length of 10 µm, and the longest measuring ∼13 µm. These results confirmed the biofunctionalization of silk nanofibers with NGF along with their availability and bioactivity for growing cells. Since a similar ‘biological sensor’ is not available for CNTF, we assumed and later confirmed with neuronal cultures, that this factor was similar to NGF in not being released but biologically active and available in/on the silk fibroin fibers.

### 3 Dorsal root ganglion (DRG) cells cultured on fibroin nanofibers

**Figure 4:**
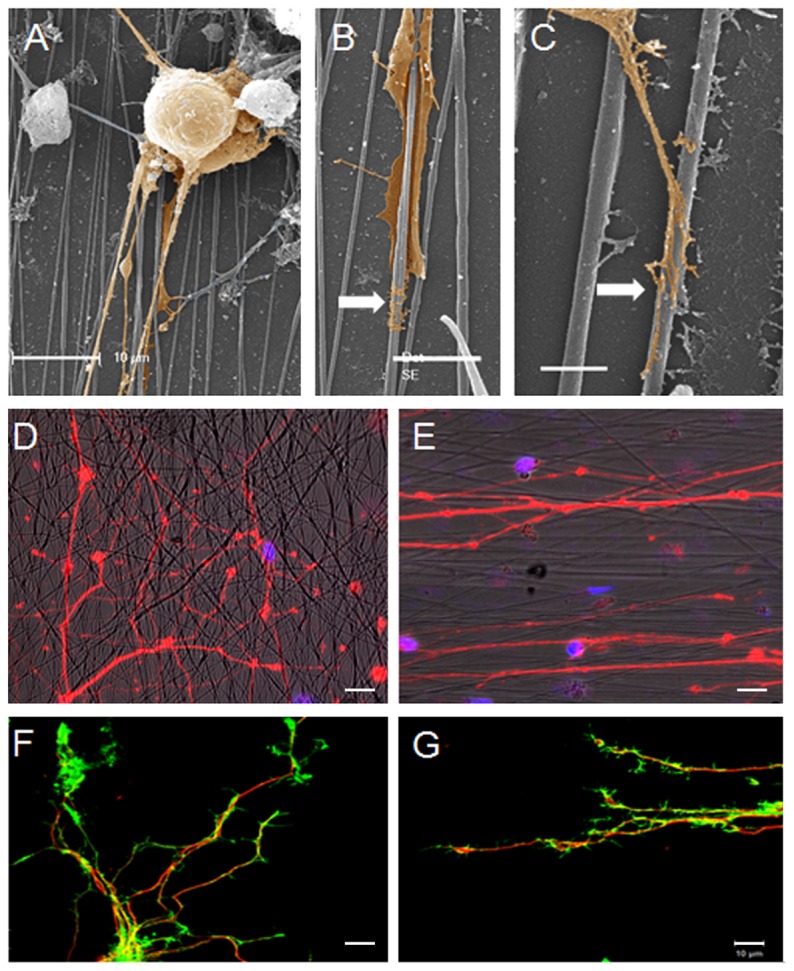
DRG neurons adhesion and growth on electrospun fibroin nanofibers. SEM observation of DRG cells adhering on nanofibers (A) and establishing tight contact (B and C). BIII tubulin staining of neurons growing on random (D) and aligned (E) nanofibers. BIII tubulin (red) and actin (green) of neuron growth cones on random (F) and aligned (G) nanofibers. Image acquisition performed after 5 days of culture. Scale bars: A 10µm, B 5µm, C 2µm, D, E, F and G 10µm. Images A, B and C have been recolored for sake of clarity.

With the perspective of guiding peripheral nerve regeneration, the ability of the silk fibers to allow adhesion and guide the growth of primary sensory neurons from rat DRG was assessed. Cells grown for 5 days on the nanofibers displayed good adherence and neuronal morphology (round cell body and sprouting neurites, Figure 4A) with some cells wrapping themselves up around nanofibers (arrow, Figure 4B) and also establishing tight contact with nanofibers (arrow, Figure 4C). When cultured on unoriented nanofibers, neurons grew randomly with neurites extending in all directions (Figure 4D) unlike neurons seeded on the aligned nanofibers which grew in the same orientation as the fibers (Figure 4E). This was highlighted by the neuron growth cone morphology and orientation on aligned fibers (Figure 4G) in contrast to those extending on randomly-oriented nanofibers (Figure 4F). Fibroin nanofibers thus supported good alignment for primary sensory neurons and orientation of neuron growth.

### 4 DRG cells culture on functionalized fibroin nanofibers

**Figure 5:**
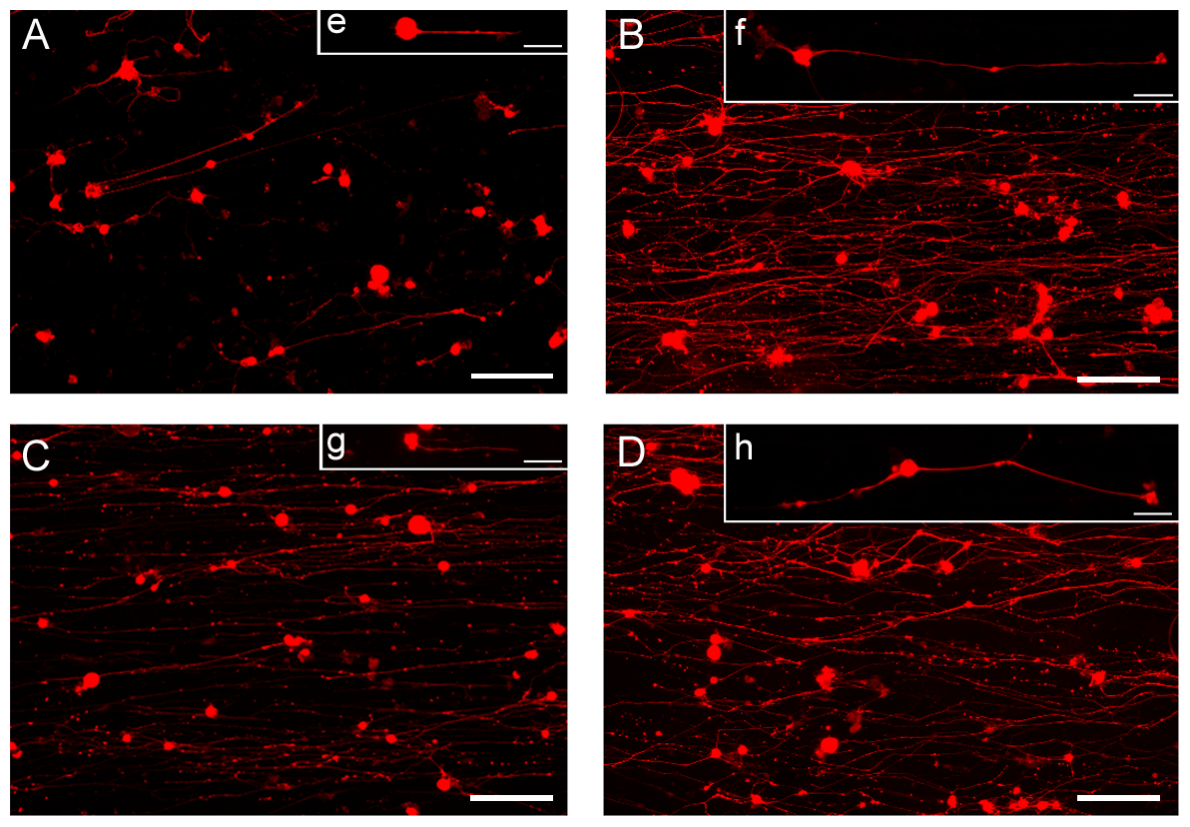
Bioactivity of functionalized electrospun fibroin nanofibers on DRG neurons growth. DRGs cells were seeded on either unfunctionalized (A and e), NGF-functionalized (B and f), CNTF (C and g) or NGF and CNTF-functionalized nanofibers (D and h) and neurons were stained with anti BIII tubulin antibody. Fluorescence images were acquired after 5 days of culture (A, B, C and D). Higher magnification of individual growing neurons (e, f, g and h) were acquired 3 days after seeding, to prevent overlapping of neurons currently observed after a longer time in culture. For details see material and methods. Scale bars: A, B, C, D: 100µm; e, f, g, h: 20 µm.

Promoting DRG cell growth using fibroin nanofibers functionalized with growth factors was then assessed. After 3 and 5 days of culture, the effect of fibroin fibers functionalized with NGF, CNTF and both of these factors were compared to non-functionalized control fibers (Figure 5). Five days after seeding on control fibers, neurons displayed short neurite outgrowth when compared to NGF- and NGF/CNTF- multifunctionalized fibers. In these functionalized systems an important neural tubulin staining was observed, confirming the neuron growth (Figure 5 A, B and D). Figure 5 e, f, g and h show a typical neuron grown for 3 days on control fibers, NGF-functionalized, CNTF-functionalized and NGF/CNTF-multifunctionalized fibers, respectively. In order to quantify the biological effects of the functionalized silk fibers, two morphological parameters were assessed: the average length of neurites and the average number of neurites per cell. A third parameter was the glial cells/neurons ratio (Table 1). After 3 days of culture, neurons grown on control fibers had an average of 1 neurite with a mean length of 60 µm and the glial cells/neurons ratio observed was 1.22. NGF-functionalized fibers stimulated neurite outgrowth with more than a 3× increase in the mean neurite length compared to the unfunctionalized nanofibers (192.5 µm and 60.1 µm, respectively). Neither the average number of neurites per neuron nor the glial cells/neuron ratios with NGF-functionalized nanofibers were statistically different from the fibroin controls.

**Table 1:** Quantification of nanofibers fibers bioactivity on dissociated DRGs cell culture.

	Nb of neurites per neuron	Neurites length [µm]	Glia/neurons ratio
**Fibroin**	0.97±0.3	60.1±28.3	1.22±0.22
**NGF**	1.5±0.6	192.5±80.3*	1.42±0.4
**CNTF**	0.72±0.6	78.9±39.5	3.02±0.49^#^
**NGF/CNTF**	1.08±0.7	124.4±45.9*	3.20±1.2^#^

Average number of neurites per cell, average neurites length and glia/neurons ratio after 3 days of culture on control fibroin nanofibers, NGF-, CNTF- and NGF/CNTF-functionalized nanofibers; Data ± SEM from 3 independent experiments for each condition and at least 90 neurons have been analyzed per condition. * and # indicate statistically significant difference from the fibroin condition for neurites length and glial cells/neuron ratio, respectively. For details see Material and methods. Nb: number.

With the CNTF-functionalized nanofibers, a significant increase in glial cell/neuron ratio was observed compared to the control fibers and NGF-functionalized fibers (3.02, 1.42 and 1.22, respectively). There was also no significant stimulation of neurite outgrowth or sprouting. Cells grown on the multifunctionalized NGF/CNTF-fibers displayed greater neurite length and an increased glial cell/neurons ratio compared to the fibroin nanofibers alone (124 µm and 3.2, respectively).

### 5 Organotypic dorsal root ganglia culture on multifunctionalized fibroin nanofibers

**Figure 6:**
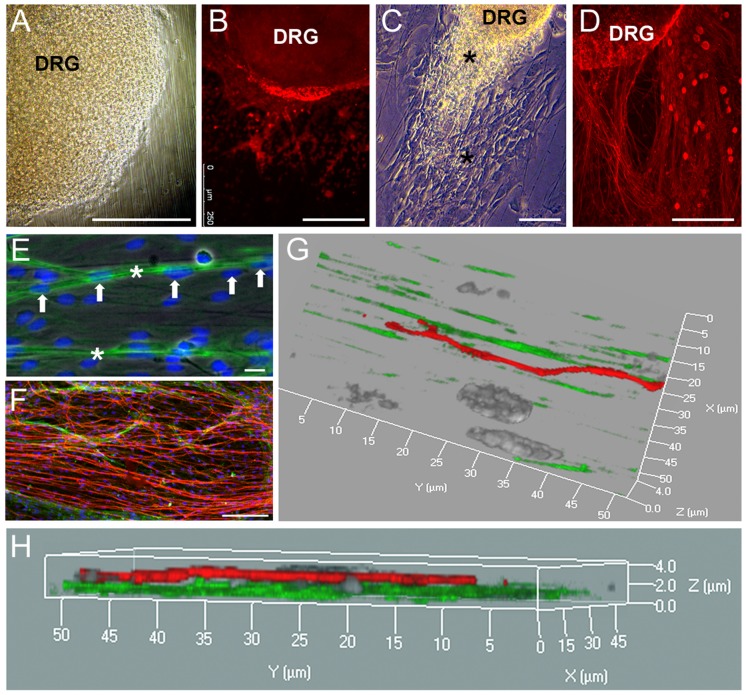
Bioactivity of multifunctionalized nanofibers in organotypic DRG culture. Fœtal DRG were seeded on unfunctionalized (A, B) or multifunctionalized (C–H) nanofibers. Images were acquired after 12 days (A, C) and 4 weeks (B, D–H) of culture. Fluorescence (B, D, E, F) and scanning laser confocal microscopy (G, H) images were obtained as described in material and methods. DRG neural tubulin was stained with anti-BIII tubulin (red), glial cells actin was stained with phalloidin (green) and nuclei with DAPI (blue). Scale bars: A, B, C, D: 250 µm; E: 10µm and G: 50µm.

Dorsal root ganglia contain both neurons and non-neuronal cells so the expectation was that DRG organotypic cultures would mimic the *in vivo* impact of the neurotrophic factor-functionalized nanofibers, including Schwann cell migration and axonal growth. DRGs were seeded on either unfunctionalized or multifunctionalized (NGF and CNTF) fibroin nanofibers in order to combine the neuron and glial cells stimulating effect of NGF and CNTF, respectively. Bright field observations of the ganglia showed a lack of cell growth and/or migration with the control fibers (Figure 6A), whereas cells grew and migrated outside the ganglion, along the fiber orientation on the multifunctionalized nanofibers over 12 days (Figure 6C, stars). After 3 weeks of culture, weak neurite outgrowth was achieved without growth factors (Figure 6B), in contrast to the mutlifunctionalized fibers where strong neural tubulin staining was found, supporting the significant neurite sprouting from the DRGs (Figure 6D). As mentioned above, dorsal root ganglia contain both neural and non-neuronal cells, including Schwann/glial cells. Higher magnifications of actin-stained glial cells showed nuclei aligned along the multifunctionalized nanofibers, attesting glial cell migration (arrows and stars, Figure 6E). The concomitant observation of both neural tubulin and glial actin staining emphasizes the importance of ‘axons rays’ with surrounding actin fibers (Figure 6F). Laser scanning confocal microscopy images highlight the fine organization of glial cells and neurons growing from the DRGs. After one month in culture the nanofibers supported aligned glial cell migration based on the actin staining and the axonal extensions moving forward above the Schwann cells bundles (Figures 6G and H).

### 6 Development of a 3D multifunctionalized nanofiber-based tube

**Figure 7:**
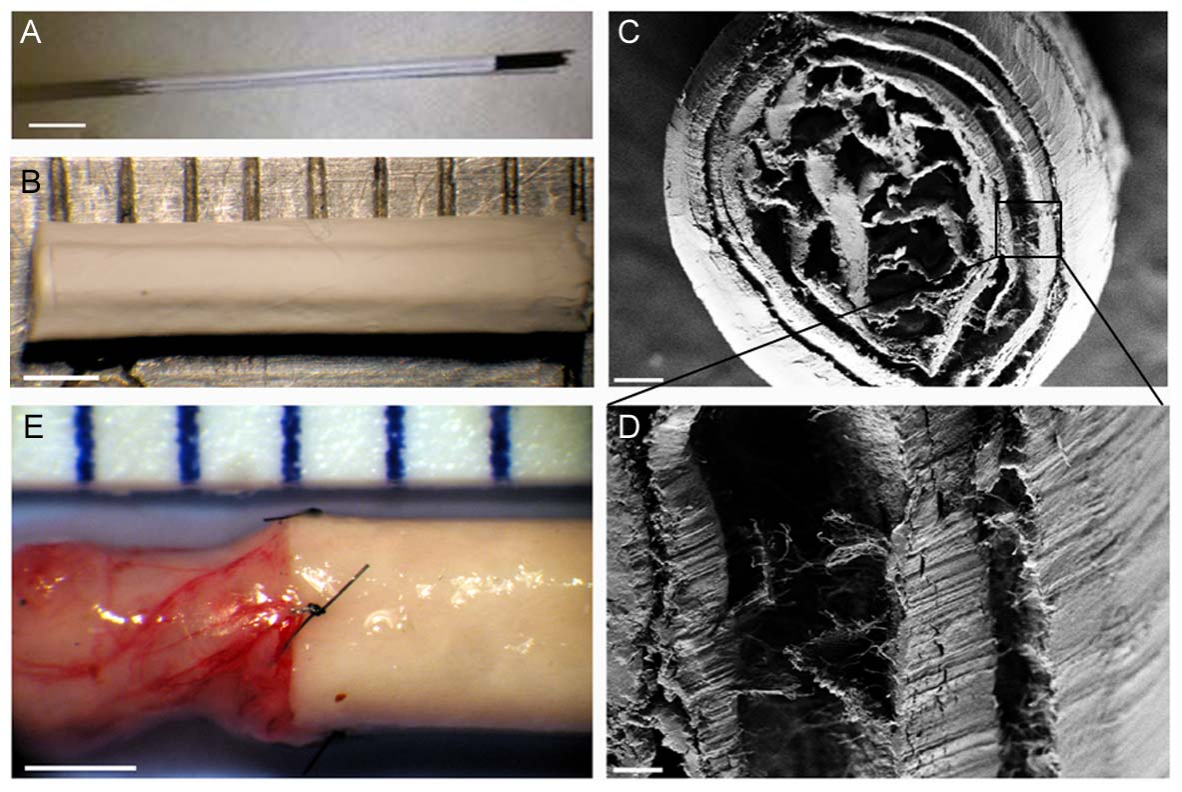
Development of a multifunctionalized nanofiber-based tube for *in vivo* implantation. Fibroin scaffold is collected at the end of the electrospinning process and rolled up on teflon sticks (A), higher magnification of the designed fibroin tube (B), SEM observation of a cross section of the fibroin tube (C), higher magnification highlighting the alignment of nanofibers inside the tube (D) and nanofiber-based tube sutured to a rat sciatic nerve (E). Scale bar: 1 cm A), 1 mm B and E), 200µm C) and 30µm D). For details see material and methods.

With the aim of producing a 3D scaffold suitable for surgical implantation in mammals, a multifunctionalized nanofiber-based tube was realized. To get close to the structure of an acellular peripheral nerve, the 2D nanofibers scaffold was rolled up using small sticks (Figure 7A) conferring tubular structure to the scaffold (Figure 7B). After removing the sticks, cross section and SEM observations depicted numerous small channels inside the tube, which also displayed well-aligned nanofibers (Figures 7 C and D). Figure 7E shows the suture of a nanofiber-based tube to a rat sciatic nerve for future *in vivo* studies.

## Discussion

The restoration of nerve pathways is a prerequisite for successful regeneration in the PNS in which glial cells play a key role during axonal regeneration [32]. Following lesion-induced Wallerian degeneration, Schwann cells start to proliferate, forming longitudinal cell strands which provide indispensable pathways for guided axonal regrowth, called bands of Bungner [33].

### 1 Fiber alignment and cell growth

Aligned fibrous scaffolds are useful in replicating the supportive guidance of some molecules and help the proper rearrangement of the required cellular environment. For a specific tissue type such as peripheral nerves where axons, glia and perineurium are aligned parallel to each other, the recapitulation of this precise nerve tissue structure is the main challenge while supporting regeneration. Besides mimicking the biomechanical supporting effect of the ECM, the alignment of electrospun nanofibers in a scaffold can also guide the migration of glial cells and extension of neuronal processes [34].

Brunetti *et al* [35] showed that neurons sense nanoscale roughness and that nanorough surfaces (Ra> 35 nm) lead to the loss of polarity and to a cytoskeleton which is not functionally organized. Thus the roughness of electrospun nanofibers (Ra =  6 nm) in our experiments is well suited for neuron adherence and growth. This is confirmed by the dissociated DRG cell growth assays in which the fibroin fibers alone promoted slight neurite extensions even in minimal medium (Figure 4). It has been recently observed that silk fibers are suitable substrates to also support CNS neuron growth, probably due to the mechanical properties acting as guidance cues to promote neurite elongation even in absence of selective growth factors [17].

### 2 Biofunctionalization

There are currently at least four methods for incorporating bioactive molecules into electrospun scaffolds: physical adsorption, covalent attachment, electrospinning with a co-axial spinneret and addition of bioactive molecules to the electrospinning solution. Covalent binding can be problematic for large molecules (MW of NGF is 27 kDa and CNTF is 23 kDa) and in order to control the final concentration of growth factors, we selected the addition of bioactive molecules to the electrospinning solution, in which growth factors are attached to the scaffold mainly through electrostatic interactions. We expected a burst release of around 20% of the factors, as is often reported from electrospun fibers [36]–[37], but we did not detect any release in our experiments. For the NGF electrospun fibers, ionic interactions could explain the lack of release as silk is negatively charged (pI 4.3) and NGF (pI 9.3) is positively charged [38]. Also, the molecular weight of commercially available NGF (27 kDa) and CNTF (23 kDa) may impede diffusion and also lead to immobilization of these proteins. These phenomena likely impacted the lack of release of NGF and CNTF from fibroin electrospun fibers. Xu *et al.* have shown that bovine serum albumin (BSA) or myoglobin release was influenced by the increase in hydrophilicity of electrospun fibrous polylactic-co-glycolic acid (PLGA) scaffolds [39].

Moreover, ELISA results showing a lack of NGF release are in accordance with the PC12 bioassays on the NGF-functionalized fibers, displaying NGF-stimulated neurites extension which is dependent on contact between nanofibers and the PC12 cells. We hypothesize that tight contacts between neurons and nanofibers observed with SEM reflect potential areas allowing growth factor uptake by neurons. Therefore, multifunctionalization of electrospun silk fibers represents an advantage compared to the local application of growth factors as it protected the factors from rapid degradation and prevented uncontrolled release.

### 3 Bioactivity and complementary effect of growth factors

This set of experiments allowed the determination of individual roles of the two growth factors as well as their synergy in terms of neural responses.

Soluble NGF plays a significant role in establishing the architecture of the peripheral nervous system during development, as well as in regeneration following axon injury, but it was only reported twice that immobilized NGF may also be bioavailable and bioactive [31], [40]. In our setup, the NGF “on demand” availability is key for adequate neuron growth from the perspective of nerve regeneration, as it is known that the dose and duration of NGF administration can determine the extent of recovery and response following peripheral nerve injury [41].

The observation in the present study that glial cell migration and stimulation with CNTF-containing nanofibers is of particular interest since Schwann cells produce a large repertoire of biochemical factors known to support axon regeneration [42]. Despite survival-promoting effects, the role of CNTF in normal adult neuronal trophic support remains unresolved and it has been speculated that CNTF may act as an “injury factor”, being released by glial cells in response to an injury [43]. Accordingly, following nerve injury, CNTF protein expression is more highly correlated with the reinnervation activity of Schwann cells than the S100 protein, and CNTF knockout mice have impaired ability to recover from a sciatic nerve crush injury [44], [45]. Moreover, it has recently been shown that CNTF acts as a chemo attractant and participates in the recruitment of endogenous progenitors during myelin repair in the central nervous system of rodents [46]. The putative role of CNTF in peripheral nerve regeneration has been emphasized by the study of Xu *et al.* in which CNTF was delivered by focal injection in a gap-silicone chamber following sciatic nerve sectioning [47]. The results suggested that CNTF promoted axon regeneration through Schwann cell migration and monocyte infiltration [41].

Numerous studies have shown that Schwann cells play an important part in biomaterial-guided peripheral nerve regeneration, albeit always seeded inside scaffolds [48]–[52].

It has also been shown that Schwann cells can migrate along longitudinal channels in a scaffold, but they were seeded before the whole DRGs were [53]. Keilhoff *et al.* also demonstrated that implanted SC adhered, survived in a collagen type I/III nerve conduit and had formed nerve-guiding columns of Büngner [54].

It is thus clear that both viable Schwann cells and structural parameters are prerequisites for successful axonal regrowth and nerve regeneration, but contrary to previously cited cell-based strategies, the present multifunctionalized nanofibers avoided the need for pre-seeding cells, which should be an advantage for surgical applications.

Furthermore, the growth rate of regenerating axons in vivo [55] and the slow and tunable degradation rate of silk-based scaffolds [56] make our nanofiber-based tube specifically relevant for peripheral nerve guided regeneration [57].

The design of this multifunctionalized nanofiber-based tube presenting multichannels provides a biomimetic organization similar to the perineurium and epineurium of peripheral nerves. This structure is easily handled by surgeons and can be sutured to the nerve. Altogether our material should create a favorable microenvironment combining a physical scaffold together with specific regenerative biochemical cues allowing peripheral nerve reconstruction.

## Conclusion

There are many obstacles that prevent successful nerve regeneration after injury in the peripheral nerve environment. One problematic area that needs to be further explored is how to appropriately combine the effects of multiple trophic factors in a complementary manner using biofunctionalized electrospun materials. The present results demonstrate the possibility to simulate neurite outgrowth with a system that combines growth support, directionality, guided glial cell migration and neurotrophic stimulation with a multifunctional biomaterial. Further studies will focus on the evaluation of these new materials in an *in vivo* model of sciatic nerve regeneration.

## References

[1] Waller A (1850) Experiments on the section of the glossopharyngeal and hypoglossal nerves of the frog, and observations of the alterations produced thereby in the structure of their primitive fibres. Philos Trans R Soc Lond Biol 140: 423–429.

[2] Stoll G, Jander S, Myers RR (2002) Degeneration and regeneration of the peripheral nervous system: from Augustus Waller's observations to neuroinflammation. J Peripher Nerv Syst 7: 13–27. 11939348 10.1046/j.1529-8027.2002.02002.x

[3] Biazar E, Khorasani MT, Montazeri N, Pourshamsian K, Daliri M, et al (2010) Types of neural guides and using nanotechnology for peripheral nerve reconstruction. Int J Nanomedicine 5: 839–852. 21042546 10.2147/IJN.S11883 PMC2963930

[4] Midha R (2006) Emerging techniques for nerve repair: nerve transfers and nerve guidance tubes. Clin Neurosurg 53: 185–90. 17380750

[5] Campbell WW (2008) Evaluation and management of peripheral nerve injury. Clinical Neurophysiol 119: 1951–1965. 10.1016/j.clinph.2008.03.018 18482862

[6] Nectow AR, Marra KG, Kaplan DL (2012) Biomaterials for the development of peripheral nerve guidance conduits. Tissue Eng Part B Rev 18: 40–45. 21812591 10.1089/ten.teb.2011.0240 PMC3262974

[7] Krick K, Tammia M, Martin R, Höke A, Mao HQ (2011) Signaling cue presentation and cell delivery to promote nerve regeneration. Curr Opin Biotechnol 22: 741–746. 21531127 10.1016/j.copbio.2011.04.002

[8] Neal RA, Tholpady SS, Foley PL, Swami N, Ogle RC, et al (2012) Alignment and composition of laminin-polycaprolactone nanofiber blends enhance peripheral nerve regeneration. J Biomed Mater Res Part A 100A: 406–423. 10.1002/jbm.a.33204 PMC3550006 22106069

[9] Wang CY, Liu JJ, Fan CY, Mo XM, Ruan HJ, et al (2012) The effect of aligned core-shell nanofibres delivering NGF on the promotion of sciatic nerve regeneration. J Biomater Sci Polym Ed. 212: 167–184. 10.1163/092050610X545805 21192836

[10] Robson RM (1985) Silk composition, structure and properties. In: Lewin M, Pearce E, editors. Fiber Chemistry Handbook of Science and Technology.Marcel Dekker, New York.

[11] Altman GH, Diaz F, Jakuba C, Calabro T, Horan RL, et al (2003) Silk-based biomaterials. Biomaterials 24: 401–416. 12423595 10.1016/s0142-9612(02)00353-8

[12] Yang Y, Zhao Y, Gu Y, Yan X, Liu J, et al (2009) Degradation behaviors of nerve guidance conduits made up of silk fibroin in vitro and in vivo. Polymer Degradation and Stability 94: 2213–2220.

[13] Pérez RA, Won JE, Knowles JC, Kim HW (2013) Naturally and synthetic smart composite biomaterials for tissue regeneration. Adv Drug Deliv Rev 65: 471–496. 22465488 10.1016/j.addr.2012.03.009

[14] Liu W, Thomopoulos S, Xia Y (2012) Electrospun nanofibers for regenerative medicine. Adv Healthc Mater 1: 10–25. 23184683 10.1002/adhm.201100021 PMC3586336

[15] Ashammakhi N, Nderu A, Nikkola L, Wimpenny I, Yang Y (2008) Advancing tissue engineering by using electrospun nanofibers. Regen Med 3: 547–574. 18588476 10.2217/17460751.3.4.547

[16] Vidal G, Blanchi T, Mieszawska AJ, Calabrese R, Rossi C, et al (2013) Enhanced cellular adhesion on titanium by silk functionalized with titanium binding and RGD peptides. Acta Biomater 9: 4935–4943. 22975628 10.1016/j.actbio.2012.09.003 PMC3508072

[17] Wittmer CR, Claudepierre T, Reber M, Wiedemann P, Garlick JA, et al (2011) Multifunctionalized electrospun silk fibers promote axon regeneration in central nervous system. Adv Funct Mater 21: 4202–4213. 22844266 10.1002/adfm.201190103 PMC3404853

[18] Lewin GR, Barde YA (1996) Physiology of the neurotrophins. Annu Rev Neurosci 19: 289–317. 8833445 10.1146/annurev.ne.19.030196.001445

[19] Thoenen H, Barde YA, Edgar D (1981) The role of nerve growth factor (NGF) and related factors for the survival of peripheral neurons. Adv Biochem Psychopharmacol 28: 263–273. 7010938

[20] Friedman B, Scherer SS, Rudge JS, Helgren M, Morrisey D, et al (1992) Regulation of ciliary neurotrophic factor expression in myelin-related Schwann cells in vivo. Neuron 9: 295–305. 1497895 10.1016/0896-6273(92)90168-d

[21] Rende M, Muir D, Ruoslahti E, Hagg T, Varon S, et al (1992) Immunolocalization of ciliary neuronotrophic factor in adult rat sciatic nerve. Glia 5: 25–32. 1531807 10.1002/glia.440050105

[22] Kang SS, Keasey MP, Cai J, Hagg T (2012) Loss of neuron-astroglial interaction rapidly induces protective CNTF expression after stroke in mice. J Neurosci 32: 9277–9787. 22764235 10.1523/JNEUROSCI.1746-12.2012 PMC3403837

[23] Lang EM, Schlegel N, Reiners K, Hofmann GO, Sendtner M, et al (2008) Single-dose application of CNTF and BDNF improves remyelination of regenerating nerve fibers after C7 ventral root avulsion and replantation. J Neurotrauma 25: 384–400. 18373486 10.1089/neu.2007.0396

[24] Jin HJ, Fridrikh SV, Rutledge GC, Kaplan DL (2002) Electrospinning Bombyx mori silk with poly(ethylene oxide). Biomacromolecules 3: 1233–1239. 12425660 10.1021/bm025581u

[25] Jose RR, Elia R, Firpo MA, Kaplan DL, Peattie RA (2012) Seamless, axially aligned, fiber tubes, meshes, microbundles and gradient biomaterial constructs. . J Mater Sci Mater Med 23: 2679–95. 22890517 10.1007/s10856-012-4739-7 PMC3493794

[26] Hu X, Shmelev K, Sun L, Gil ES, Park SH, et al (2011) Regulation of silk material structure by temperature-controlled water vapor annealing. Biomacromolecules 12: 1686–96. 21425769 10.1021/bm200062a PMC3090511

[27] De Groot P and Deck L (1994) Optical measurements and sensors for the process industries. Proc. SPIE 2248, pp.101–104.

[28] Malin SA, Davis BM, Molliver DC (2007) Production of dissociated sensory neuron cultures and considerations for their use in studying neuronal function and plasticity. Nat Protoc 2: 152–160. 17401349 10.1038/nprot.2006.461

[29] Rajaraman R, Rounds DE, Yen SP (1974) A scanning electron microscope study of cell adhesion and spreading in vitro. Exp Cell Res 88: 327–339. 4426334 10.1016/0014-4827(74)90248-1

[30] Teng K K, Greene L A (1994) Cell biology: a laboratory handbook. In: Celis J E, editor. San Diego, Calif: Academic Press. pp.218–224.

[31] Lee JY, Bashur CA, Milroy CA, Forciniti L, Goldstein AS, et al (2012) Nerve growth factor-immobilized electrically conducting fibrous scaffolds for potential use in neural engineering applications. IEEE Trans Nanobioscience 11: 15–21. 21712166 10.1109/TNB.2011.2159621 PMC4648550

[32] Frostick SP, Yin Q, Kemp GJ (1998) Schwann cells, neurotrophic factors, and peripheral nerve regeneration. Microsurgery 18: 397–405. 9880154 10.1002/(sici)1098-2752(1998)18:7<397::aid-micr2>3.0.co;2-f

[33] Bungner OV (1891) Über die Degenerations- und Regenerationsvorgänge am Nerven nach Verletzungen. Beitr Pathol Anat 10: 321–87.

[34] Chew SY, Mi RF, Hoke A, Leong KW (2007) Aligned Protein-Polymer Composite Fibers Enhance Nerve Regeneration: A Potential Tissue-Engineering Platform. Adv Funct Mater 17: 1288–1296. 18618021 10.1002/adfm.200600441 PMC2447933

[35] Brunetti V, Maiorano G, Rizzello L, Sorce B, Sabella S, et al (2010) Neurons sense nanoscale roughness with nanometer sensitivity. Proc Natl Acad Sci U S A 107: 6264–6469. 20308580 10.1073/pnas.0914456107 PMC2851967

[36] Schneider A, Wang XY, Kaplan DL, Garlick JA, Egles C (2009) Biofunctionalized electrospun silk mats as a topical bioactive dressing for accelerated wound healing. Acta Biomater 5: 2570–2578. 19162575 10.1016/j.actbio.2008.12.013 PMC2810481

[37] Sahoo S, Ang LT, Goh JC, Toh SL (2010) Growth factor delivery through electrospun nanofibers in scaffolds for tissue engineering applications. J Biomed Mater Res A 93: 1539–1550. 20014288 10.1002/jbm.a.32645

[38] Uebersax L, Mattotti M, Papaloïzos M, Merkle HP, Gander B, et al (2007) Silk fibroin matrices for the controlled release of nerve growth factor (NGF). Biomaterials 28: 4449–4460. 17643485 10.1016/j.biomaterials.2007.06.034

[39] Xu W, Atala A, Yoo JJ, Lee SJ (2013) Controllable dual protein delivery through electrospun fibrous scaffolds with different hydrophilicities. Biomed Mater 8: 014104. 23353662 10.1088/1748-6041/8/1/014104

[40] Sandrock AW Jr, Matthew WD (1987) Substrate-bound nerve growth factor promotes neurite growth in peripheral nerve. Brain Res 1987 425: 360–363. 10.1016/0006-8993(87)90520-8 3427437

[41] Kemp SW, Webb AA, Dhaliwal S, Syed S, Walsh SK, et al (2011) Dose and duration of nerve growth factor (NGF) administration determine the extent of behavioral recovery following peripheral nerve injury in the rat. Exp Neurol 229: 460–70. 21458449 10.1016/j.expneurol.2011.03.017

[42] Pfister LA, Papaloizos M, Merkle HP, Gander B (2007) Nerve conduits and growth factor delivery in peripheral nerve repair. J Peripher Nerv Syst 12: 65–82. 17565531 10.1111/j.1529-8027.2007.00125.x

[43] Adler R (1993) Ciliary neurotrophic factor as an injury factor. Curr Opin Neurobiol 3: 785–789. 8260830 10.1016/0959-4388(93)90154-q

[44] Hiruma S, Shimizu T, Huruta T, Satou T, Hu J, et al (1997) Ciliary neurotrophic factor immunoreactivity in rat intramuscular nerve during reinnervation through a silicone tube after severing of the rat sciatic nerve. Exp Mol Pathol 64: 23–30. 9203506 10.1006/exmp.1997.2206

[45] Yao M, Moir MS, Wang MZ, To MP, Terris DJ (1999) Peripheral nerve regeneration in CNTF knockout mice. Laryngoscope 109: 1263–1268. 10443831 10.1097/00005537-199908000-00015

[46] Vernerey J, Macchi M, Magalon K, Cayre M, Durbec P (2013) Ciliary neurotrophic factor controls progenitor migration during remyelination in the adult rodent brain. J Neurosci 33: 3240–3250. 23407977 10.1523/JNEUROSCI.2579-12.2013 PMC6619230

[47] Xu JJ, Chen EY, Lu CL, He C (2009) Recombinant ciliary neurotrophic factor promotes nerve regeneration and induces gene expression in silicon tube-bridged transected sciatic nerves in adult rats. J Clin Neurosci 16: 812–817. 19289286 10.1016/j.jocn.2008.08.035

[48] Guenard V, Kleitman N, Morrissey TK, Bunge RP, Aebischer P (1992) Syngeneic Schwann cells derived from adult nerves seeded in semipermeable guidance channels enhance peripheral nerve regeneration. J Neurosci 12: 3310–3320. 1527582 10.1523/JNEUROSCI.12-09-03310.1992 PMC6575729

[49] Kim DH, Connolly SE, Kline DG, Voorhies RM, Smith A, et al (1994) Labeled Schwann cell transplants versus sural nerve grafts in nerve repair. J Neurosurg 80: 254–260. 8283264 10.3171/jns.1994.80.2.0254

[50] Levi AD, Sonntag VK, Dickman C, Mather J, Li RH, et al (1997) The role of cultured Schwann cell grafts in the repair of gaps within the peripheral nervous system of primates. Exp Neurol 143: 25–36. 9000443 10.1006/exnr.1996.6344

[51] Jesuraj NJ, Santosa KB, Macewan MR, Moore AM, Kasukurthi R, et al (2014) Schwann cells seeded in acellular nerve grafts improve functional recovery. Muscle Nerve 49: 267–276. 23625513 10.1002/mus.23885 PMC4112584

[52] Berrocal YA, Almeida VW, Gupta R, Levi AD (2013) Transplantation of Schwann cells in a collagen tube for the repair of large, segmental peripheral nerve defects in rats. J Neurosurg 119: 720–732. 23746104 10.3171/2013.4.JNS121189

[53] Bozkurt A, Deumens R, Beckmann C, Olde Damink L, Schügner F, et al (2009) In vitro cell alignment obtained with a Schwann cell enriched microstructured nerve guide with longitudinal guidance channels. Biomaterials 30: 169–179. 18922575 10.1016/j.biomaterials.2008.09.017

[54] Keilhoff G, Stang F, Wolf G, Fansa H (2003) Bio-compatibility of type I/III collagen matrix for peripheral nerve reconstruction. Biomaterials 24: 2779–2787. 12711525 10.1016/s0142-9612(03)00084-x

[55] Al-Majed AA, Neumann CM, Brushart TM, Gordon T (2000) Brief electrical stimulation promotes the speed and accuracy of motor axonal regeneration. J Neurosci. 20: 2602–2608. 10.1523/JNEUROSCI.20-07-02602.2000 PMC6772244 10729340

[56] Wang Y, Rudym DD, Walsh A, Abrahamsen L, Kim HJ, et al (2008) In vivo degradation of three-dimensional silk fibroin scaffolds. Biomaterials. (24–25): 3415–3428. 10.1016/j.biomaterials.2008.05.002 PMC3206261 18502501

[57] Tang X, Xue C, Wang Y, Ding F, Yang Y, et al (2012) Bridging peripheral nerve defects with a tissue engineered nerve graft composed of an in vitro cultured nerve equivalent and a silk fibroin-based scaffold Biomaterials. Biomaterials 33: 3860–3867. 22364696 10.1016/j.biomaterials.2012.02.008

